# Risk estimation using probability machines

**DOI:** 10.1186/1756-0381-7-2

**Published:** 2014-03-01

**Authors:** Abhijit Dasgupta, Silke Szymczak, Jason H Moore, Joan E Bailey-Wilson, James D Malley

**Affiliations:** 1 Clinical Trials and Outcomes Branch, National Institute of Arthritis, Musculoskeletal and Skin Diseases, National Institutes of Health, Room 4-1350, Bldg 10 CRC, 10 Center Drive, Bethesda, MD 20892-1468, USA; 2 Inherited Disease Research Branch, National Human Genome Research Institute, National Institutes of Health, 333 Cassell Drive, Suite 1200, Baltimore 21224MD, USA; 3 Department of Genetics, Dartmouth College, HB 7937, Dartmouth-Hitchcock Medical Center, One Medical Center Drive, NH 03756 Lebanon, USA; 4 Mathematical and Statistical Computing Laboratory, Center for Information Technology, National Institutes of Health, Bldg 12A, Room 2039, Bethesda, MD 20892-5620, USA; 5 Current address: Institute of Clinical Molecular Biology, Christian-Albrechts-University Kiel, Am Botanischen Garten 11, 24118 Kiel, Germany

**Keywords:** Consistent nonparametric regression, Logistic regression, Probability machine, Odds ratio, Counterfactuals, Interactions

## Abstract

**Background:**

Logistic regression has been the *de facto*, and often the only, model used in the description and analysis of relationships between a binary outcome and observed features. It is widely used to obtain the conditional probabilities of the outcome given predictors, as well as predictor effect size estimates using conditional odds ratios.

**Results:**

We show how statistical learning machines for binary outcomes, provably consistent for the nonparametric regression problem, can be used to provide both consistent conditional probability estimation and conditional effect size estimates. Effect size estimates from learning machines leverage our understanding of counterfactual arguments central to the interpretation of such estimates. We show that, if the data generating model is logistic, we can recover accurate probability predictions and effect size estimates with nearly the same efficiency as a correct logistic model, both for main effects and interactions. We also propose a method using learning machines to scan for possible interaction effects quickly and efficiently. Simulations using random forest probability machines are presented.

**Conclusions:**

The models we propose make no assumptions about the data structure, and capture the patterns in the data by just specifying the predictors involved and not any particular model structure. So they do not run the same risks of model mis-specification and the resultant estimation biases as a logistic model. This methodology, which we call a “risk machine”, will share properties from the statistical machine that it is derived from.

## Background

Logistic regression has been the *de facto*, and often the only, model used in the description and analysis of relationships between a binary outcome and observed features, both categorical and continuous. It is widely used both as an association model and a predictive model, to look at (a) the conditional probability of outcome, given predictors, and (b) predictor effect size estimates using conditional odds ratios. It is also widely available in software and is easy to optimize in low-dimensional problems. However, it does assume the data-generating model to be logistic, requires an explicit specification of the model, and is not scalable to the higher dimensional problems that are so common today. Good predictions and effect size estimation from a logistic model therefore require the researcher to guess at the true data generating model, and to exactly specify which predictors appear and how they interact with each other. If the model is mis-specified, both predictions and effect size estimates may be more than slightly in error. In modeling terms, the challenge is to get all the main effects and interactions (2-way and higher order) correctly specified in the model; otherwise efficient and consistent estimation is not certain.

We recently introduced the concept of a *probability machine (PM)*[1], which is simply any consistent nonparametric regression machine applied to binary or categorical outcomes. A PM produces a predicted conditional probability of success given predictors, but in a model-agnostic data-driven fashion. The idea is that, starting from binary (0/1) outcomes for each subject, a PM generates an estimated expected value for each subject, which is just the conditional probability of success for that subject given predictors. Given today’s rich data environment, a PM has several desirable properties. It can study any list of predictors (binary, categorical, continuous), requires no explicit structural specification of the model, no specification of interactions, and is scalable to very large sets of predictors, for example over a million single nucleotide polymorphisms (SNPs) in genome-wide association studies. As a practical matter, there are many well-studied families of nonparametric learning machines that are provably consistent for the regression problem–in the limit of large data the error rate converges to the Bayes error rate–and with reasonably speedy convergence. The class of nonparametric regression machines we have studied come from the machine learning literature, namely random forest regression [2] and nearest neighbor regression. Both of these have provable consistency properties under fairly general conditions [3-5]. There are of course other possible choices, like some support vector machines. In this work, we focus on random forest regression used with a {0,1} outcome and call it *a random forest probability machine (RFPM)*.

A RFPM, which is a regression random forest, is scalable to large datasets and high-dimensional problems and requires only a specification of which features are to be included in the machine rather than any explicit functional form. We have shown in our earlier work [1] that even when data is generated from a logistic model, the test set error when estimating the outcome probability of success using a RFPM often beats that using logistic regression. In this work we look at the role of logistic regression in particular and how the PM can play a similar role both in probability prediction and effect size estimation under prospective sampling schemes. We show that with data generated by a logistic model, a PM can do just as well as a correctly-specified logistic regression for both problems, and can be superior when the logistic regression is not correctly specified or inappropriate given the data generating model. We also suggest that a PM can do more than just probability and effect estimation as with logistic regression. It can also be used for descriptive discovery of interactions, among other descriptive analyses. Moreover, as probability machines are known to be consistent, the probability estimates and derived risk estimates will also be consistent. This also holds for interaction detection across any subsets of features. We expand on these themes in the following sections.

## Methods

### Conditional probability estimation

We first consider the problem of conditional probability estimation. Under prospective sampling schemes, both logistic regression (LR) and PMs can estimate the conditional probability of success given a set of features. The consistency of the LR model is established as long as the data generating mechanism is logistic, and the correct model is specified. Under model mis-specification, it is known that the LR model is no longer consistent. As practitioners know, establishing the adequacy of a particular logistic model for a data set is not easy, and no tests to check the validity of the logistic link are routinely available. We believe that any logistic regression model fit to a data set is likely to be incorrect in its specification, though in some cases we may be more confident based on other knowledge about the data generating mechanism. The RFPM, on the other hand, is known to be consistent under more general conditions [3,5] and thus can produce valid estimates of the conditional probability under a wider variety of conditions. We note here that we will use a random forest of *regression trees*, where each component tree provides a probability estimate of success (denoted in [1] as *regRF*). These individual probability estimates are then averaged across the trees to provide the regRF estimate. The random forest methodology also can provide a self-declared “probability estimate” when used as a classifier, which is nothing but the proportion of component trees which classified the result as a success. We have found in [1] that this method, there denoted by *classRF*, is far less efficient at probability estimation, and the consistency of the probability estimates thus produced has not been demonstrated in the literature.

### Simulations

We will first see how a RFPM compares to a LR when the generative model truly is logistic. We generated multiple simulation studies using binary predictors. We considered 1, 2 or 3 binary predictors with main effects and/or interactions with respect to a binary outcome, and add 7 other binary predictors with no association with the outcome. This is motivated by genomic studies where the signal is typically sparse and several genes have no association with the outcome. Details may be found in Tables 1 and 2. We will fit three models:

1. A main effects logistic regression using all available features (LR1)

2. A logistic regression with main effects and all possible two-way interactions using all available features (LR2)

3. A random forest regression using all available features (RFPM, denoted as RF in the graphs)

**Table 1:** Summary of logistic regression models used for simulation studies

**Model**	**Description**	**Conditional odds ratios**
1	3 main effects	1.3, 1.7, and 2.5
2	3 main effects and 2 interactions	1.3, 1.7, 2.5 (main effects); 2, 5 (interactions)
3	See Table 2	
4	2 main effects	1.3, 2 (main effects)
5	2 main effects + 1 interaction	1.3, 2 (main effects); 2 (interaction)

This table summarizes the structures of the logistic regression models we have used to generate data in the simulation studies in this manuscript.

**Table 2:** Structure of the model in model 3

**Stratum**	**1**	**2**	**3**	**4**	**5**	**6**	**7**	**8**
**X**_**1**_	0	1	0	0	1	1	0	1
**X**_**2**_	0	0	1	0	1	0	1	1
**X**_**3**_	0	0	0	1	0	1	1	1
**Probability**	0.300	0.176	0.563	0.391	0.563	0.096	0.794	0.836

We define 8 strata based on combinations of 3 binary predictors X_1_, X_2_ and X_3_. The conditional probability of success of the outcome Y for each of these strata is given in the table. Outcomes Y are simulated within each stratum using a binomial random number generator with probability of success given in the table.

LR1 is the usual model that is tried in most situations, since interactions are harder to estimate and require more data for adequate power and efficiency. LR2 is a model that accounts for 2-way interactions in a non-committal manner. *A priori*, we do not know which interactions are actually present, and so we will interrogate all possible such interactions. Note that this is not a fully saturated model, since that would include all higher order interactions as well. For data with a large number of available features, the LR2-type model quickly becomes unfeasible, since the number of parameters grows at the rate *p*^2^ when *p* is the number of available features. The RFPM only needs which features to include, and will consider complex interactions implicitly, as we will see. It will also not break down as quickly as *p* increases, and is used routinely with *p* as large as 100 K or 1 M or even larger; we have experience using it with over 100 million features in a genomic data set [6].

In all our simulations, we use the *randomForest* package ([7], version 4.6) in R, with tuning parameters *nodesize* (minimum size of the terminal nodes) set at 5% of the total sample size, and *mtry* equaling the number of features. We have experimented with smaller *mtry* values but have found that setting *mtry* to be the number of features gives the best results. We have also experimented with setting the number of trees in the random forest to be anywhere from 20 to 1000, and do not see much difference in the results over this span. Our reported simulations use 100 trees per random forest. We have also done simulations in Python using the random forest function from the scikits-learn Python package [8], giving similar results to the R runs, albeit at slightly slower speed. Since the results from Python are similar to the results from R, only results from R are reported here. The code to run these simulations, in either R or Python, is available upon request.

From our simulations we highlight two cases. Model 1 will consist of a binary outcome generated under a logistic model from 3 independent binary predictors with baseline success probability 0.3, conditional odds ratios of 1.2, 1.5 and 2 respectively and 7 other independent binary predictors with no association with the outcome, i.e.,

$$\mathrm{logit}\left(p\right)=\mathrm{logit}\left(0.3\right)+\log\left(1.2\right)\;X_{1}+\log\left(1.5\right)\;X_{2}+\log\left(2\right)\;X_{3}+0\;X_{4}+\ldots +0\;X_{10}$$

Model 2 is the same as model 1, except that there are interactions between *X*_*1*_ and *X*_*2*_ and between *X*_*2*_ and *X*_*3*_ with interaction odds ratios of 2 and 5, respectively. The quality of the estimated probabilities of success, conditional on the predictors, was evaluated using bias and efficiency. Bias is measured by the difference between the true conditional probability used to generate the data sets and the average individual prediction over the simulated data sets. Efficiency in predicting the individual conditional probability is measured by the width of the interval defined by the 5th and 95th percentiles of the simulated distribution of predictions for each individual.

**Figure 1:**
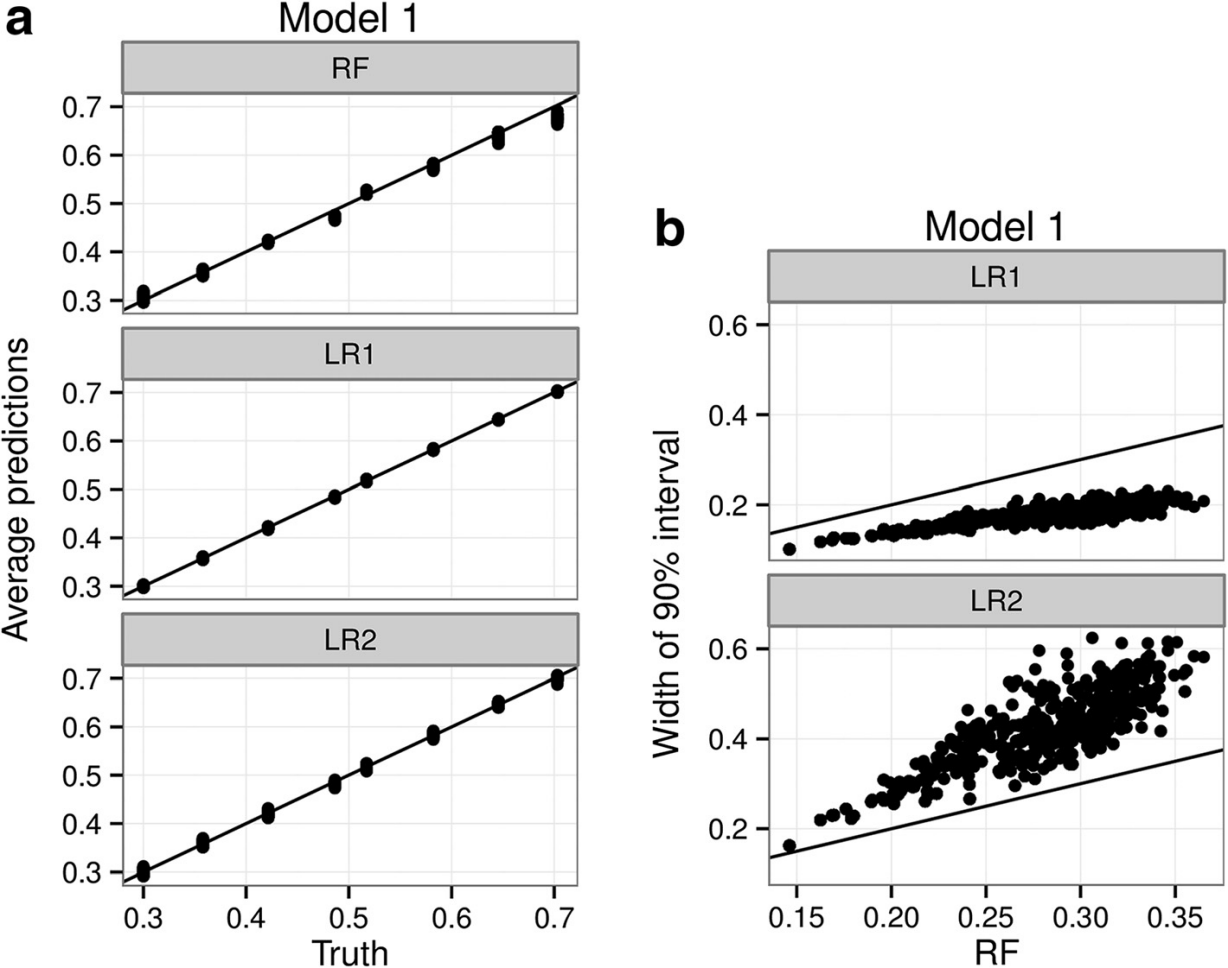
**Comparative performance of RFPM and logistic regression under a main effects model.** A main effects logistic regression model (LR1), a main effects + 2-way interaction logistic regression model (LR2) and a Random Forest Probability Machine (RF) are fit to 1000 simulated data sets under Model 1 (data with only 3 main effects under a logistic data generating model). Figure 1**(a)** Average conditional probability estimates of LR1, LR2 and RF are compared with the true probabilities in the data generating model. Figure 1**(b)** Width of the interval defined by the 5^th^ and 95^th^ percentiles of the simulation distribution of each probability target over 1000 simulations are compared between the three models that are fit.

Figure 1 shows the performance of the three fitted models under Model 1. We find that all three models provide reasonably unbiased estimation of the true probabilities, though naturally LR1 and LR2 are less biased since they are in the family of the data-generating model. We also find that LR1 is most efficient, more efficient than LR2. This is because degrees of freedom are being expended in estimating the interactions that, in this model, do not exist. The RFPM has efficiency intermediate to the two, reflecting the fact that it implicitly finds a sparse model [5], but is still not as efficient as a logistic model when the data is generated from a logistic model. This is not surprising to us, since we’re being penalized for not assuming a particular correct structure for the model.

**Figure 2:**
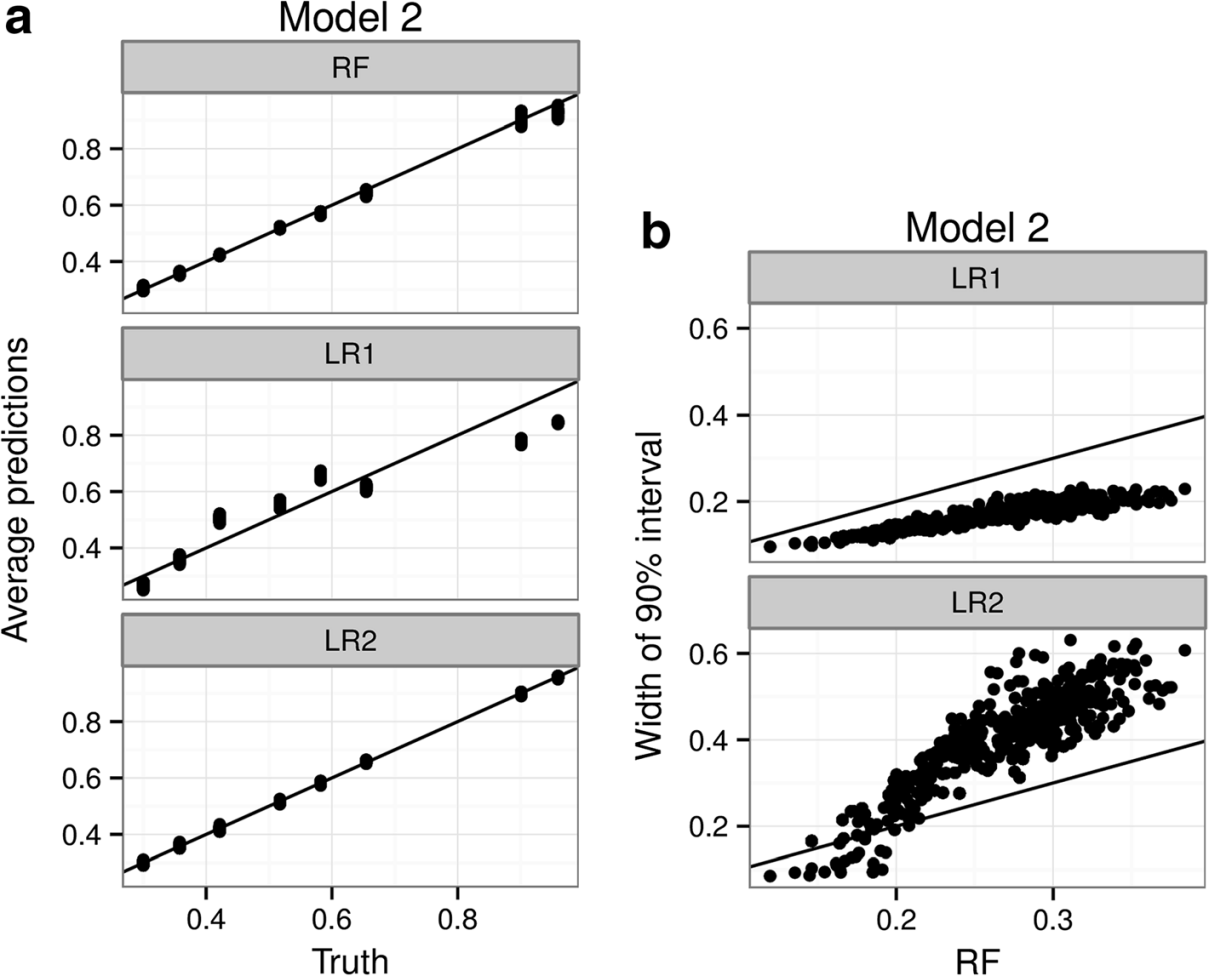
**Comparative performance of RFPM and logistic regression under an interaction model.** A main effects logistic regression model (LR1), a main effects + 2-way interaction logistic regression model (LR2) and a Random Forest Probability Machine (RF) are fit to 1000 simulated data sets under Model 2 (data with 3 main effects and 2 two-way interactions) under a logistic data generating model). Figure 2**(a)** Average conditional probability estimates of LR1, LR2 and RF are compared with the true probabilities in the data generating model. Figure 2**(b)** Width of the interval defined by the 5^th^ and 95^th^ percentiles of the simulation distribution of each probability target over 1000 simulations are compared between the three models that are fit.

Figure 2 shows the performance of the models under Model 2. Since Model 2 contains interactions that are not specified in LR1, LR1 does poorly in estimating the true probabilities. LR2 does contain these interactions, so the estimated probabilities are accurate. RFPM also provides accurate estimates of the probabilities, but without any explicit specification of any interactions. Once again the efficiency of RFPM is intermediate to LR1 and LR2, since we’re paying a penalty for nonparametric estimation but not as much as estimating multiple unnecessary parameters; RFPM still optimizes to a sparser model than LR2 implicitly. However, we would like to note that, in practice, LR1 is the usual model that is fit to data and is reported on; modeling for interactions is not routinely done specially as the number of predictors increase. Given that LR1 is nominally the model most often used, Figure 2a shows that the presence of interactions can severely bias its results. RFPM accommodates potential interactions without *a priori* specification and hence maintains good predictive performance. As an initial model for binary outcomes, RFPM provides a more robust solution with respect to model specification than the main effects logistic paradigm.

**Figure 3:**
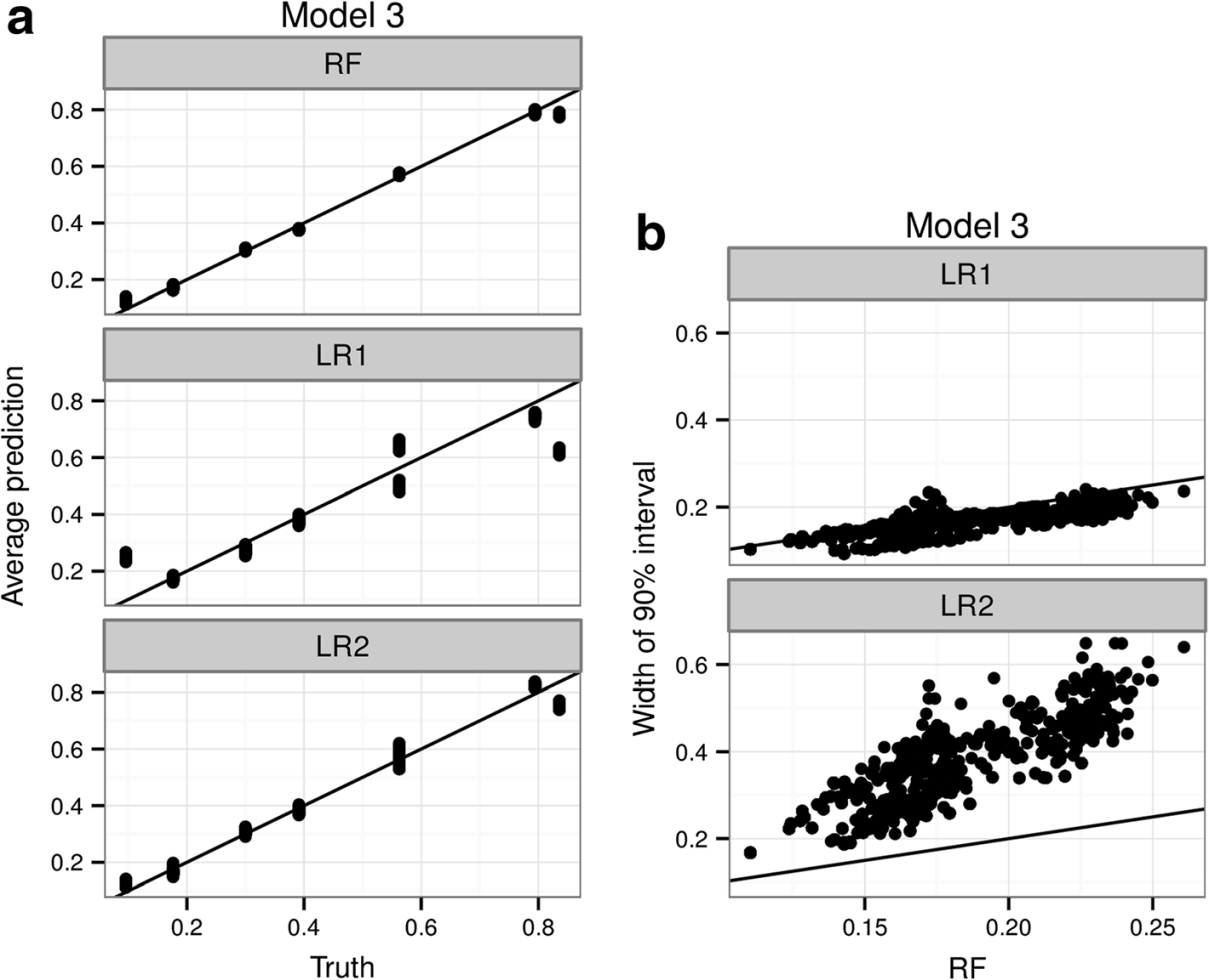
**Comparative performance of RFPM and logistic regression under a saturated model.** A main effects logistic regression model (LR1), a main effects + 2-way interaction logistic regression model (LR2) and a Random Forest Probability Machine (RF) are fit to 1000 simulated data sets under Model 3 (a saturated model). Figure 3**(a)** Average conditional probability estimates of LR1, LR2 and RF are compared with the true probabilities in the data generating model. Figure 3**(b)** Width of the interval defined by the 5^th^ and 95^th^ percentiles of the simulation distribution of each probability target over 1000 simulations are compared between the three models that are fit.

To further make the point about the sensitivity of logistic regression to model specification, we simulated data from a logistic model where each of the 8 subgroups defined by X_1_, X_2_ and X_3_ has an arbitrary conditional probability. This is in effect a saturated logistic model with a 3-way interaction term. We still keep the 7 unassociated predictors as before. As we can see in Figure 3a, predictions from both LR1 and LR2 miss the true probabilities, LR1 to a greater degree than LR2, since the generating model is not a sub model of either LR1 or LR2. The RFPM, on the other hand, more accurately estimates most of the probabilities *with no change in the model specification* compared to the modeling done for Models 1 and 2 earlier. Figure 3b shows that the RFPM is now just as efficient as LR1, and more efficient than LR2. These combined shows that RFPM is a better choice of initial model when the generative model, though logistic, is not a sub-model of the LR models being fitted.

### Counterfactual machines

Counterfactual arguments are the basis of our interpretation of regression coefficients. Regression coefficients are interpreted to be the average change in outcome when a predictor changes by one unit, *all other predictors remaining the same*. In other words, we are asking what *could* happen if we change just one factor, everything else being equal. We argue that, using probability machines, we can look directly at counterfactual outcomes in the context of binary outcomes to see the counterfactual change in success probability for each individual when one binary predictor is changed. The core idea can be extended easily to continuous outcomes and conceptually to continuous predictors, which we will present in a future manuscript.

Conceptually, we can consider two groups of individuals where each individual in one group is identical to an individual in the other group, except for the value of one feature *X*_*1*_. If this were possible, we could directly observe the changes in outcome in each person resulting from changing the value of *X*_*1*_ by merely observing their corresponding doppelganger in the other group. Such experiments are carried out regularly in, for example, comparing wild-type clonal mice versus mice where a particular gene is knocked out, while treating both groups the same. Such experiments are of course not possible in human population studies. We can, however, get a very good sense of how one’s doppelganger would behave using predictive models. We train two predictive models, one for individuals with *X*_*1*_ = 0 and one for individuals with *X*_*1*_ = 1. Each captures the feature landscape, so to speak, and its relationship with the outcome in each subgroup. Now, if we predict the outcome of an individual with *X*_*1*_ = 1 using the predictive model trained on the *X*_*1*_ = 0 subgroup, it would be as if we supplanted this individual into the feature landscape of the *X*_*1*_ = 0 group, and the prediction would be reflective of the relationship between this feature landscape and the outcome, preserving all the other feature information about this individual. In other words, we can mimic the behavior of this individual’s conceptual doppelganger, and the difference between an individual’s observed outcome and their predicted outcome using a predictive model trained on the other group would be the counterfactual effect of *X*_*1*_ on that individual.

We can operationalize the description above using RFPMs. Suppose we want to predict the counterfactual outcomes for each individual when the value of the binary predictor *X*_*1*_ is changed. We split the dataset into two subgroups *D*_*0*_ and *D*_*1*_ based on whether *X*_*1*_ is 0 or 1. We now train identically specified RFPMs on each subgroup, calling the trained RFPMs *PM*_*0*_ and *PM*_*1*_ respectively. Now, for binary data, we can’t directly observe the probability of success, but we can estimate it based on our models. For an individual with *X*_*1*_ = 0, their “observed” probability of success would be the prediction from *PM*_*0*_ and their counterfactual probability of success would be their prediction from *PM*_*1*_. We can similarly compute the “observed” and counterfactual probabilities of success of an individual with *X*_*1*_ = 1 using predictions from *PM*_*1*_ and *PM*_*0*_ respectively. Thus, for each individual, we can compute under the RFPM model a probability *p*_*0*_ and a probability *p*_*1*_ of success under the conditions *X*_*1*_ = 0 and *X*_*1*_ = 1 respectively.

Conceptually there is nothing in this operationalization that limits us to RFPMs or even PMs. You could do the same exercise using a logistic model as well. However, a logistic model would just give back estimates reflective of the chosen model structure provided it is the correct model; it would merely be a self-fulfilling exercise. If the logistic model is mis-specified, this exercise will give you estimates that are different from what the model provides. PMs provide non-parametric estimates without a particular model structure, so this exercise can help find dependency patterns in the data without assumptions about particular links or particular structural constraints like linearity.

### Risk machines: generating risk effect estimates using probability machines

Logistic regression thrives on providing risk estimates, in particular conditional odds ratio estimates, with respect to predictors within the model. Using the concept of counterfactual machines described earlier, generating estimates of different risk estimates becomes straightforward using probability machines. In fact, since we are able to get estimates of both the observed and counterfactual probabilities of success *p*_*0*_ and *p*_*1*_, we can actually estimate any function of them, not just the odds ratio which is a constraint of the logistic or multiplicative model. For example we can estimate conditional risk differences and risk ratios *at an individual level* as well as odds ratios. More precisely, for each subject in the study population, we can compute

1. the risk difference (RD): *p*_1_ - *p*_0_

2. the risk ratio (RR): *p*_1_/*p*_0_

3. the odds ratio (OR): *p*_1_(1 - *p*_0_)/*p*_0_(1 - *p*_1_)

We can compute group-specific estimates of each of these functions by averaging (mean or median) the individual estimates over the members of the group; the overall conditional estimates or main effects estimates are obtained by averaging over the entire study. Note that since the estimation targets the feature-specific counterfactual probabilities per subject, we are free to choose any function of them for our risk estimates. We call this method generally the “two-machine method”, since we need to train 2 machines, one on each subgroup defined by the predictor of interest *X*_*1*_.

Often we are not interested in the overall main effect but in subject-specific effects, which can lead to discovery as well as estimation of interactions. In fact, this is probably the more frequent case. That is, the assumption that the effect of a feature is unaffected by all other features–a pure main effects model–needs to be tested before it is believed. Unfortunately computational difficulties in assessing complex interactions over a large number of features under a logistic regression paradigm have made the main effects model the de facto standard rather than something to be validated. Our scheme allows us to easily obtain odds ratio estimates, or other risk function estimates, for subgroups defined by a second feature or a set of features by merely averaging the individual odds ratio estimates over each subgroup. This would then enable a very easy computation of the interaction odds ratio. In this paper, we present a second method for interaction estimation that directly leverages the counterfactual argument.

Consistency of each machine on its defining data set implies consistency of the risk estimates. Discrete features in the data with more than the two levels [Yes, No] lead to finitely more machines, yet the new calculations are straightforward. Features with continuous values could be studied by binning the exposure data, but this approach too often imposes an unacceptable loss of information and so requires further study.

### Simulations

We followed the simulation set-up described earlier, and consider scenarios with only main effects. Main effects odds ratios for each simulated data set were computed directly from each logistic regression model (LR1 and LR2); for the RFPM, subject-specific odds ratio estimates were obtained using two-machine counterfactual machines for each predictor, and the main effect odds ratio estimates were obtained by averaging over the individual odds ratio estimates. We report the results of the simulation study using Model 1, which has 10 independent binary predictors of which three have non-null main effects and no interactions between the predictors.

**Figure 4:**
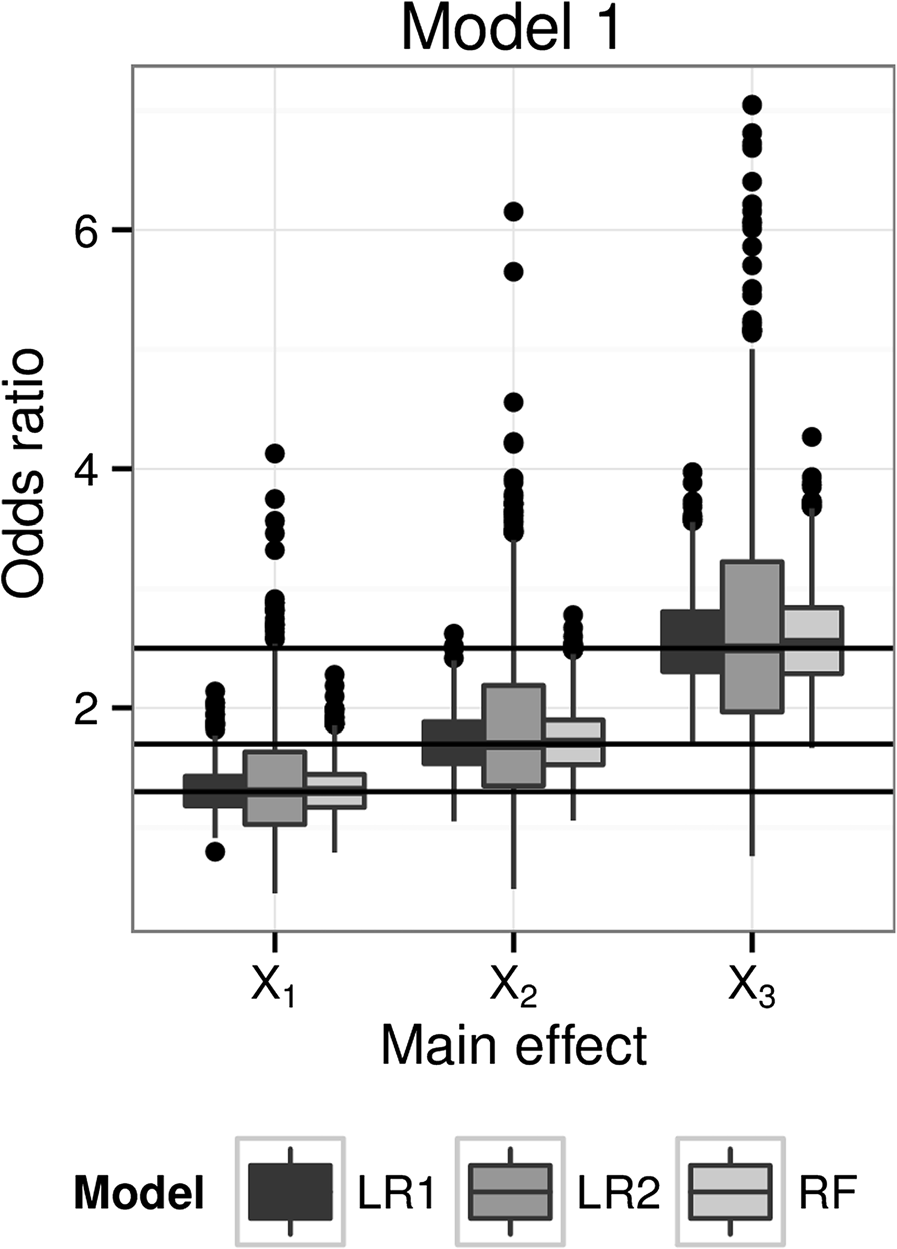
**Simulation-based distributions of odds ratio estimates using RFPM and logistic regression.** Simulated distributions of the conditional odds ratios for *X*_*1*_,* X*_*2*_, and *X*_*3*_ under Model 1 (3 main effects) estimated using a main effects logistic regression model (LR1), a logistic regression model with main effects and all two-way interactions (LR2) and a risk machine using RFPM and the “two-machine method”. The solid lines represent the true main effects present in the data.

The simulated distributions for the estimated odds ratios for each method are shown in Figure 4. We see that all the procedures evidently produce unbiased estimates, since medians of estimated odds ratios were very close to the true values (shown by the horizontal lines). In terms of variability the RFPM model is quite competitive with the LR1 model, which is the true and correct generative model. The LR2 model is typically more variable since it in effect pays a penalty for having to estimate all the two-way interactions on the same sample.

The main message here is that the two-machine counterfactual machine method can reproduce the true odds ratios from the simulation in an unbiased manner, and have efficiencies not much worse than the logistic main effects model, which is the data generating model. A logistic model which is mis-specified for the data generating model produces both inaccurate predictions and biased effect estimates, even if the correct predictors are included. Moreover, it is difficult to assess whether the model is mis-specified without further modeling and testing, a fact that is often unaccounted for in deriving inference from the final model. The RFPM model where the correct predictors are included accounts for the patterns in the data to provide accurate predictions and individual effect size estimates. Aggregate estimates of main effects and interactions and exploration of whether interactions are present can be done based on a single modeling run. The advantage that the risk machine method has is that it is not constrained by having to guess the data-generating model. In fact, in the simulation setting in Figure 3, where each of 8 predictor subgroups has a unique success probability, generated by a fully saturated model, the risk machine can in fact estimate the subgroup-specific odds ratios accurately by using an appropriate number of counterfactual machines (in this case, 2^3^ = 8 machines) or by averaging the individual odds ratio estimates over the appropriate subgroups from a single machine run.

**Figure 5:**
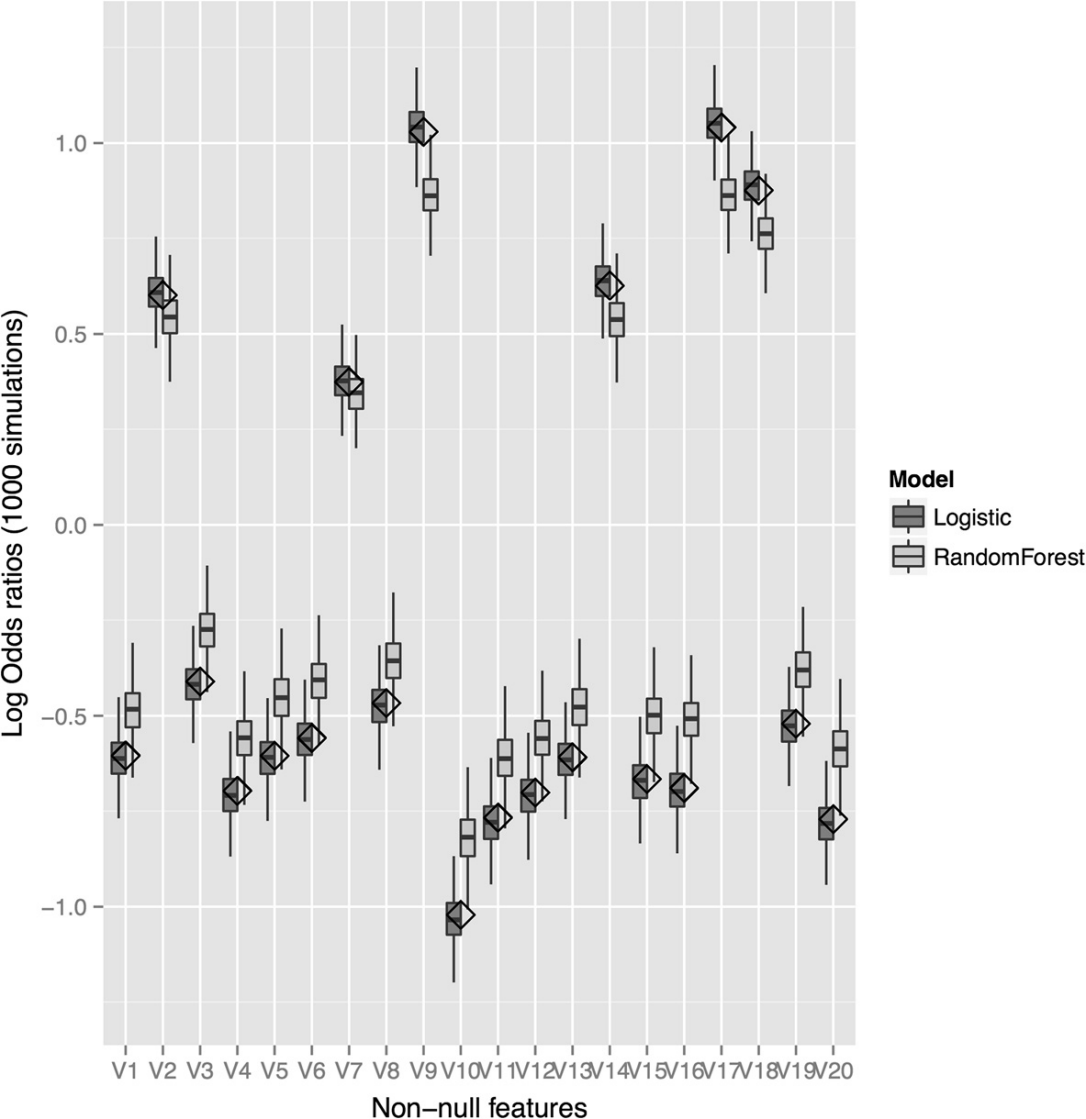
**Simulation-based distributions of odds ratio estimates from RFPM and logistic regression with 100 features.** Simulated distributions of the conditional odds ratios for features with non-null association with the outcome under the simulation model. Under this model, 20 features out of 100 have non-null associations with conditional odds ratios simulated from a N(0.7,0.2) distribution, then multiplied randomly by -1 or 1. The data is generated from a logistic regression model. We find that RFPM estimates are attenuated to the null, whereas logistic regression estimates tend to be biased away from the null. Diamonds denotes the true coefficients under the simulation model.

We have looked at how this strategy scales to larger feature sets. We have simulated 10000 observations under a logistic model with 100 features, where 20% of the features have non-zero regression coefficients simulated from a N(0.7,0.2) distribution that are then randomly multiplied by -1 or 1. We find that the RFPM risk estimates for the features that have non-null associations with outcome tend to be attenuated to the null, whereas the estimates from logistic regression tend to be biased away from the null (Figure 5). We believe that this is because, with a larger number of predictors, some of the probability machines do not have enough training data to generate good predictions. The distribution of risk estimates corresponding to all the predictors are in Additional file 1: Figure S1a (logistic regression) and Figure S1b (RFPM). We are continuing research to understand the mechanics of this behavior and strategies to improve performance.

### Interaction detection and estimation

#### Interaction estimation

It is sometimes thought that nonparametric statistical learning machines cannot accurately estimate effect sizes. As shown above using the two-machine method, consistency of the probability machine, and therefore of the risk machine, demonstrates otherwise. It is effective as a practical method for main effects odds ratio estimation, given logistic regression data over binary predictors. Of course the multiple machine method for risk effect estimation is not restricted to logistic regression data, and can be applied to any regression problem with binary outcomes.

We now consider the problem of interactions. We will start be describing estimation of multiplicative interactions as are seen in the logistic model context, for binary predictors. We introduce a “4-machine method” analogous to the earlier 2-machine method to estimate interaction effects. This works as follows. For estimating the interaction effect due to two binary predictors X_1_ and X_2_, each taking values in {0,1}, parse the data into four groups defined by the four possible values of X_1_ and X_2_, that is the four subsets of the data defined by {X_1_, X_2_} = {(0, 0), (0, 1), (1, 0), (1, 1)}. Fit a probability machine to each group separately. Note that within each subgroup the values for {X_1_, X_2_} remain fixed, so the machine will not use the specific values for either feature. The separate machines are distinctly constructed. They don’t see each other or the data for the other groups: the separate model predictions for the probability of success in the outcome are therefore conditional on the particular combinations of the predictors {X_1_, X_2_}. We now estimate the probability of success of each individual using each of the 4 machines, arriving at a vector of probabilities (*p*_00_, *p*_01_, *p*_10_, *p*_11_) for each individual. One of these represents the observed probability and the others represents counterfactual probabilities under the other (X_1_,X_2_) combinations. It is now straightforward to compute the interaction ratios for each individual, which is the ratio of the odds ratios *p*_11_(1 - *p*_10_)/(1 - *p*_11_)*p*_10_ and *p*_01_(1 - *p*_00_)/(1 - *p*_01_)*p*_00_. The overall odds ratio can be obtained by averaging these individual odds ratios over the study population. This is in fact exactly analogous to the interaction effect estimated in a logistic regression, apart from the log transformation applied to the odds ratios that is standard in logistic regression.

We note here that we need to run 4 machines to estimate each interaction, in addition to an overall machine that could be used to identify strongly predictive features. This is obviously burdensome in many current contexts. Our philosophy is not to estimate all possible interactions, but to identify particular “interesting” interactions based either on biology, previous results, or the predictive power of the features based on a global run. We note here that creating 4 subgroups may produce subgroups with a wide range of sample sizes even with moderately unbalanced predictors. Working with unbalanced predictors is not a problem when using regression machines since we are estimating the expectation of a binary outcome. We have run several simulations to satisfy ourselves that this theoretical result holds. However, due to potential loss of sample size in each stratum, there may be the need to adjust the parameters of the machine, such as the terminal node size in a random forest, to accommodate the practical issues of fitting the model accurately.

#### Intuitive model-free interaction detection

We now present a method for exploring the presence of interactions from a single machine run. This method cannot be used directly for estimation, though the derived estimates might be reasonably close to the truth, since accounting for counterfactual effects is not done. We present this method using RFPM as our representative probability machine, though other machines can be used just as effectively.

We will use RFPM to discover interactions visually and intuitively without invoking parametric models *using a single global machine run*. We have seen that fitting a RFPM to binary outcome regression data provides consistent estimates of the probability *Prob(Y = 1|X)*. A single run only gives predictions of the conditional probabilities and not any counterfactual predictions–it is not a counterfactual machine.

Consider our usual scenario where we have a binary outcome *Y*, whose expectation is predicted using several binary predictors *X*_*1*_*, X*_*2*_*, . . . , X*_*p*_, and where we wish to explore the interaction of *X*_*1*_ and *X*_*2*_ on *Y*. Let each predictor take values in {0,1}. We first fit a single *RFPM* to the data, and obtain estimates *Prob*(*Y* =1 | *X*) of the conditional probability of *Y* = 1. Next, compute the average predicted probabilities conditional on the four possible combinations over (*X*_*1*_*, X*_*2*_) and define

$$\begin{array}{lll} p_{00} & = & {\mathrm{average}}_{X_{3},\ldots ,X_{p}}\mathrm{logit}(\mathrm{Prob}\left(Y|X_{1}=0,X_{2}=0,X_{3},\ldots ,X_{p}\right))\\ p_{10} & = & {\mathrm{average}}_{X_{3},\ldots ,X_{p}}\mathrm{logit}(\mathrm{Prob}\left(Y|X_{1}=1,X_{2}=0,X_{3},\ldots ,X_{p}\right))\\ p_{01} & = & {\mathrm{average}}_{X_{3},\ldots ,X_{p}}\mathrm{logit}(\mathrm{Prob}\left(Y|X_{1}=0,X_{2}=1,X_{3},\ldots ,X_{p}\right))\\ p_{11} & = & {\mathrm{average}}_{X_{3},\ldots ,X_{p}}\mathrm{logit}(\mathrm{Prob}\left(Y|X_{1}=1,X_{2}=1,X_{3},\ldots ,X_{p}\right)) \end{array}$$

Note that these are the subgroup-specific averages from a single probability machine run and not counterfactual computations. An interaction plot is created by displaying these four values against values of *X*_*1*_*.* As in classical analysis of variance, we check if the line joining *p*_*01*_ and *p*_*11*_ is parallel to the line joining *p*_*00*_ and *p*_*10*_. This is a check for multiplicative interactions. For additive interactions, we replace the averages over the logit-transformed probabilities above with the averages of the probability estimates themselves.

### Simulations

Consider a logistic model with outcome *Y* and ten binary predictors *X*_*1*_*,…,X*_*10*_ each with P(*X*_*i*_ *= 1*) = 0.3, with main effects odds ratios of 1.3 and 2 corresponding to *X*_*1*_, and *X*_*2*_, and 1 for the rest. Call this Model 4. We add interaction odds ratios of 2 corresponding to the predictor pair (*X*_*1*_,X_2_) to Model 4 and call it Model 5. We generate 1000 data points under each model and repeat 1000 times.

**Figure 6:**
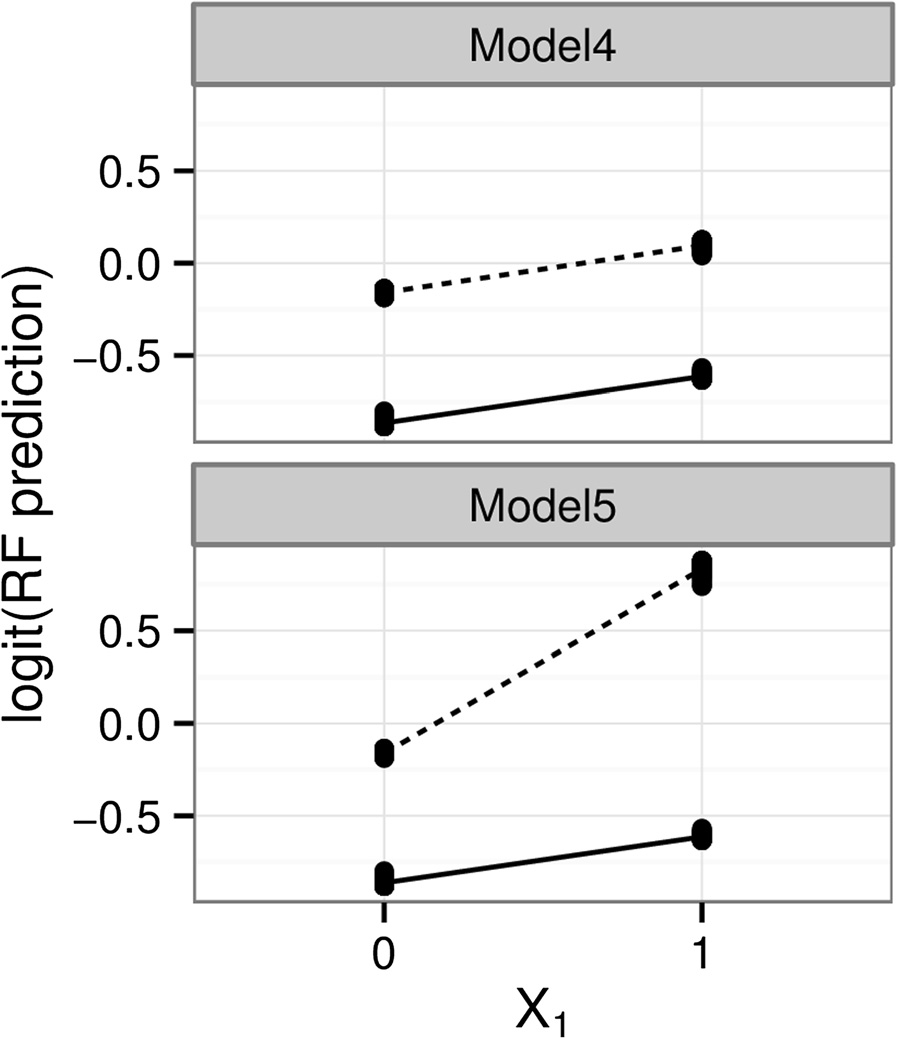
**Interaction plots.** Interaction plots for the (*X*_*1,*_*X*_*2*_) interaction under Model 4 (no true interaction) and Model 5 (Interaction odds ratio = 2). We find that the lines for Model 4 are reasonably parallel, whereas in Model 5 they are not, indicating presence of multiplicative interaction. Note the y-axis are logit-transformed probabilities. The solid line represents the subgroup with *X*_*2*_ = 0 and the dotted line represents the subgroup with *X*_*2*_ = 1.

We first generate interaction plots as described above for the (*X*_*1,*_*X*_*2*_) interaction effect under each of the two models for a particular simulated data set (Figure 6). The figures indicate that there is no interaction effect between *X*_*1*_ and *X*_*2*_ in Model 4 but some multiplicative interaction in Model 5. These results were consistent across the simulated data sets.

We can compute the classical contrast T = *p*_*11*_*-p*_*10*_*-p*_*01*_ *+ p*_*00*_ from the fitted machine. If the lines are parallel, i.e., no multiplicative interaction, T should be 0. This contrast can be used as an indicator to detect the presence of an interaction over pairs of features. In Model 4, T = 0.004 (true value = 0) and in Model 5 it is 0.747 (true value = 0.693) for the (*X*_*1*_,*X*_*2*_) interaction.

**Figure 7:**
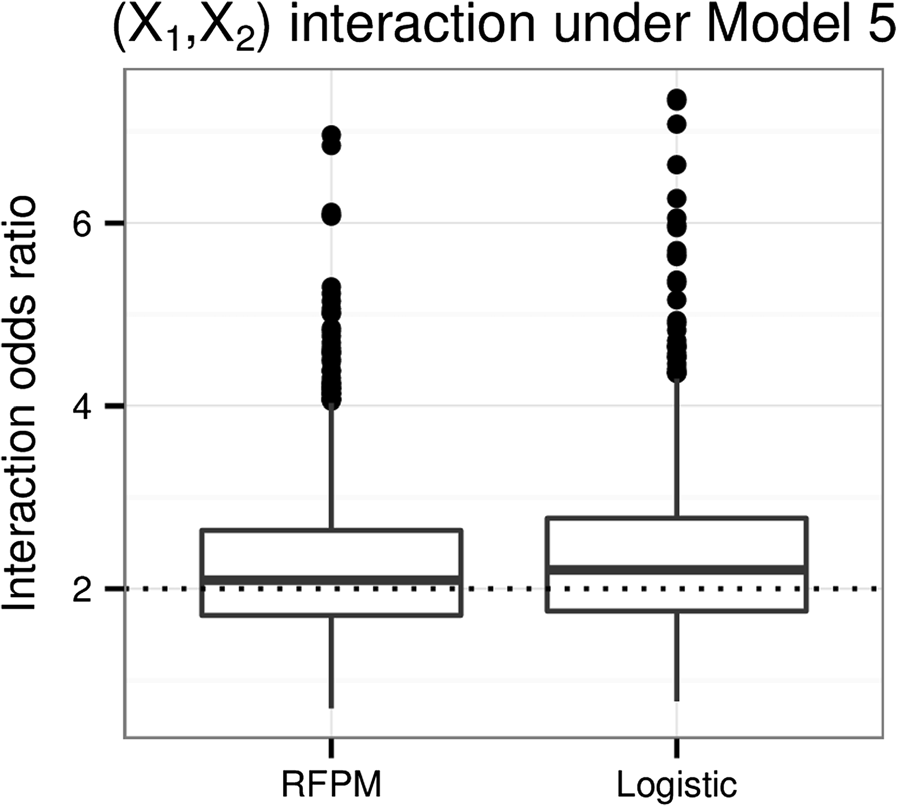
**Simulation-based distribution of estimates of multiplicative interaction using RFPM and logistic regression.** Simulation distributions for the estimates of the (*X*_*1*_,*X*_*2*_) multiplicative interaction under Model 5 using a RFPM risk machine approach (RFPM) and a logistic regression model with main effects and all possible two-way interactions (Logistic). The dotted line represents the true interaction odds ratio of 2.

We can use the 4-machine method described earlier to estimate the interaction effect of (*X*_*1*_,*X*_*2*_) in Model 5. Figure 7 shows the results of the simulation study, where the box plots represent the simulated distribution of the estimates of the (*X*_*1,*_*X*_*2*_) interactions in the data generating model using the logistic regression model LR2 (main effects + all two-way interactions) as used earlier, and the 4-machine RFPM-based method, denoted RFPM. We find that the logistic regression estimate is biased slightly upwards, whereas the RFPM-based estimates are accurate and have similar precision as the logistic regression based estimates. These results show that RFPM-based methods, and indeed any consistent probability machine, can be used within the 4-machine methodology to accurately detect and estimate multiplicative interaction effects when they truly exist.

The discovery method leveraging interaction plots and the linear contrast can be used to scan pairs of predictors to quickly find potentially interacting predictors, and the 4-machine estimation method can be used to estimate the interaction effects for those interacting predictors. We can visualize the sets of potentially interacting predictors using a heat map, where each axis has the set of predictors of interest and the color is based on the magnitude of the linear contrast statistic T. Note that we can just as easily investigate *additive interaction* under the same general scheme with the exact same RFPM as before, with no additional modeling runs; this is due to the fact that we are predicting the counterfactual probabilities in a nonparametric, model-free manner and so can estimate any particular function of them immediately. We are no longer limited by the logistic model’s constraint of multiplicative interaction estimation.

The extension of this methodology to categorical predictors is straightforward. Plausible and efficient extensions to continuous predictors are under study.

## Conclusions

We believe there are several advantages to the learning machine approach for risk effect estimation. This approach leverages directly the concept of counterfactual estimates and consistent predictive models to get predictions of the counterfactual probabilities. First, since these predicted counterfactual probabilities are generated by probability machines which are consistent, the individual counterfactual probability estimates should also be consistent, within the respective contexts X = 0 and X = 1. Obtaining good estimates of these individual probabilities grants us the flexibility of directly estimating different risk effect functions, such as risk differences, risk ratios and odds ratios, that are all based on the standard counterfactual argument. More precisely, these risk machine estimates are nonparametric estimates of the population effects and are not influenced by assumptions made in modeling as is essential for using classical logistic regression. This property also allows us to empirically understand the nature of the effect in the additive, multiplicative or other scale, rather than having to begin with an assumption of multiplicative interaction, as when using logistic regression.

Second, one cannot typically assume that data is generated from purely main effects. Our simulations show that if interactions are suspected, then *RFPM* is an accurate and more efficient choice for estimating the conditional probabilities, and then for estimating the odds ratios, than is the exploratory logistic regression, *LR2*, which includes all two-way interactions. Of course, interactions need not be limited to two-way interactions, and including higher order interactions in a logistic framework quickly increases the number of parameters to be estimated, hence further reducing efficiency. The model-free risk machine approach also allows us to freely consider complex nonlinear interactions, as when the odds ratios or risk ratios change nonlinearly with another continuous predictor.

Third, the risk machine *RFPM* intrinsically incorporates higher order interactions in its tree and forest based probability estimation and so can smoothly accommodate risk estimation over higher order interactions, without having them inserted as features in the analysis before the machine is applied to the data.

Fourth, note that for all the experiments described, the specification of the *RFPM* machine was identical, in that we only tell the machine what the predictors are and identify the outcome: nothing more is assumed as part of any model building approach. We can be entirely agnostic to the generative mechanism of the data while invoking a nonparametric risk machine such as *RFPM* and still end with good estimation and prediction. Such is not the case for a logistic regression approach unless it estimates from a correct and fully specified model.

The risk machine approach, therefore, is an efficient and practical way to interrogate data with binary outcomes, free of the usual hazard of model misspecification. Effectively the researcher does not need to validate or calibrate a parametric model before efficient and unbiased risk estimation can be studied, and the data analytic energy can be directed at consideration of what makes functional sense for risk estimation across all the features.

## Abbreviations

PM: Probability Machine; RFPM: Random Forest Probability Machine; LR: Logistic regression; OR: Odds ratio; RR: Relative risk; RD: Risk difference.

## Competing interests

The authors declare that they have no competing interests.

## Authors’ contributions

AD helped conceive the problem, develop the solution, provided insight into counterfactual machines, carried out the simulation studies, developed the methods for interaction detection and drafted the manuscript. SS helped with the simulation studies and helped draft the manuscript. JHM helped with defining the problem and drafting the manuscript. JEBW helped with developing the methods and drafting the manuscript. JDM helped conceive the problem, develop the solution, discovered the idea of counterfactual machines, and helped draft the manuscript. All authors read and approved the final manuscript.

## Supplementary Material

Additional file 1: Figure S1 These figures show the distribution of conditional odds ratios for all the features in the simulation model described in Figure 5. This model has 100 features and a sample size of 10,000, and 1000 simulated data sets were generated following a logistic model. 20 features have non-null association with the outcome, with the logistic coefficients (log-odds ratios) simulated from a N(0.7,0.2) distribution and then randomly multiplied by -1 or 1. **Figure S1a** shows the results from fitting logistic regressions to the simulated data sets. **Figure S1b** shows the results from fitting RFPM to the simulated data sets. Diamonds denotes the true log-odds ratios under the simulation model. Click here for file
