# Supplementary figures and images for: Risk estimation using probability machines
Source: BioData Min. 2014 Mar 1;7:2. doi: 10.1186/1756-0381-7-2 (PMC4015350; doi:10.1186/1756-0381-7-2)

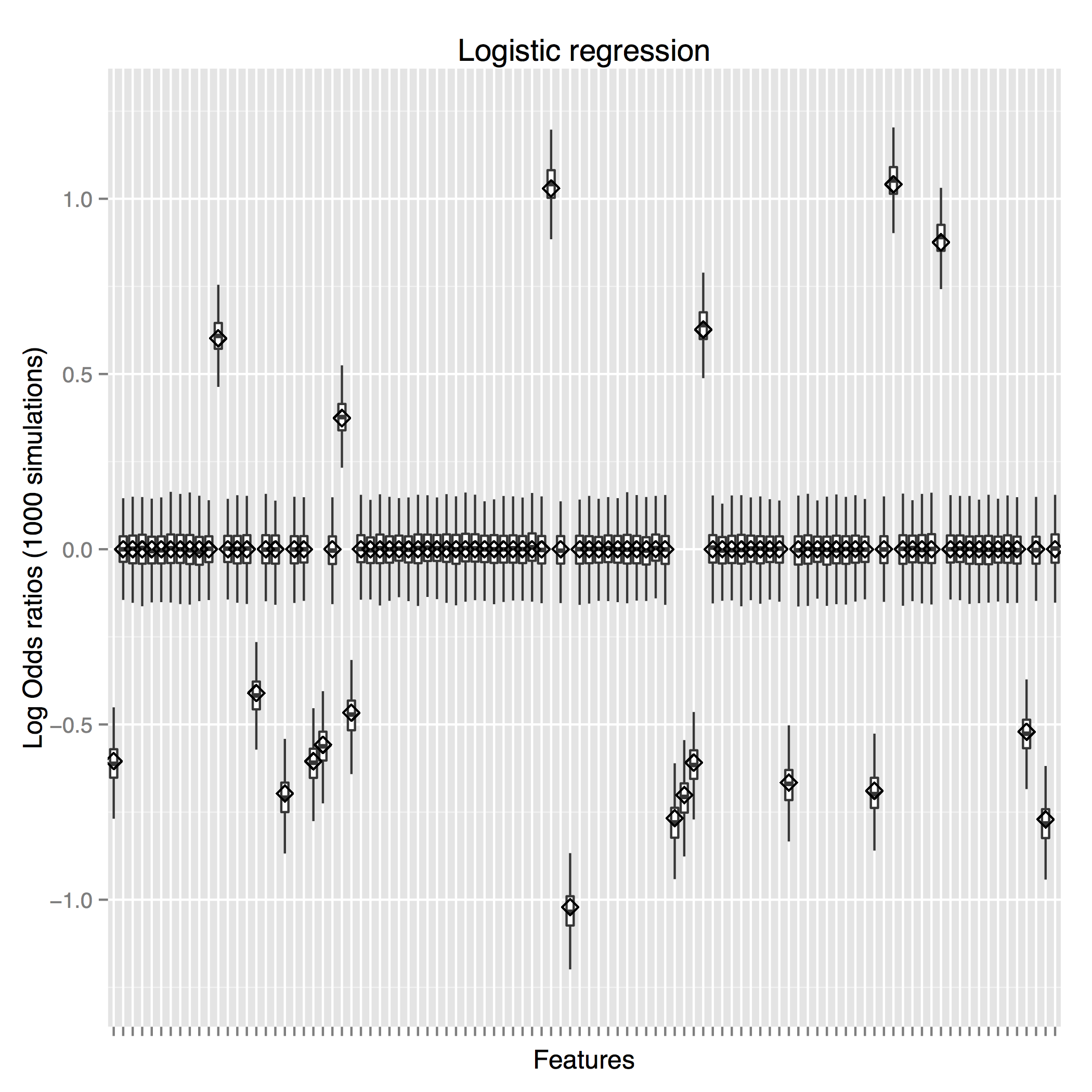

Supplement: Additional file 1: Figure S1 — These figures show the distribution of conditional odds ratios for all the features in the simulation model described in Figure 5. This model has 100 features and a sample size of 10,000, and 1000 simulated data sets were generated following a logistic model. 20 features have non-null association with the outcome, with the logistic coefficients (log-odds ratios) simulated from a N(0.7,0.2) distribution and then randomly multiplied by -1 or 1. Figure S1a shows the results from fitting logistic regressions to the simulated data sets. Figure S1b shows the results from fitting RFPM to the simulated data sets. Diamonds denotes the true log-odds ratios under the simulation model. [file 1756-0381-7-2-S1.zip › 7341464451022578_add1.tiff]

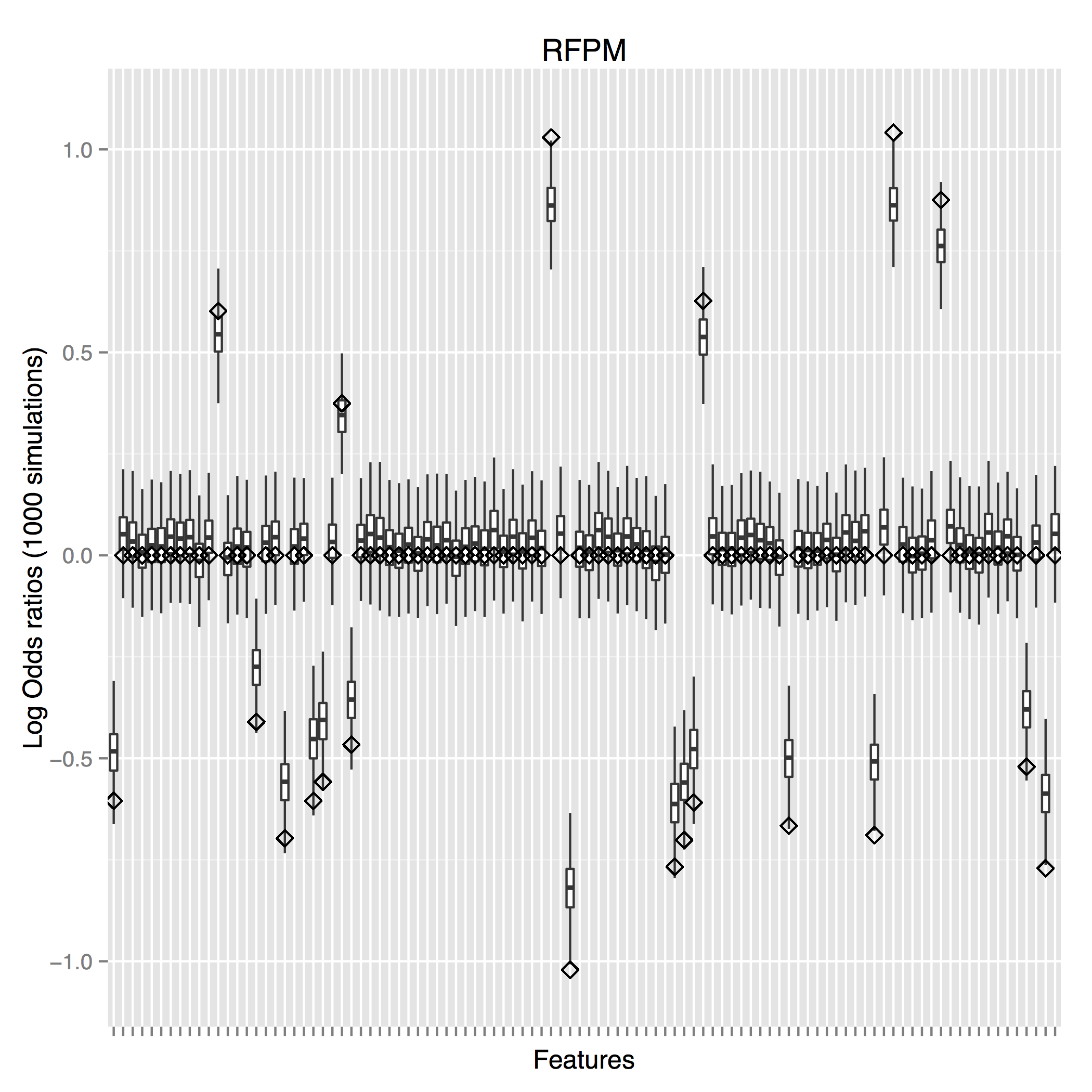

Supplement: Additional file 1: Figure S1 — These figures show the distribution of conditional odds ratios for all the features in the simulation model described in Figure 5. This model has 100 features and a sample size of 10,000, and 1000 simulated data sets were generated following a logistic model. 20 features have non-null association with the outcome, with the logistic coefficients (log-odds ratios) simulated from a N(0.7,0.2) distribution and then randomly multiplied by -1 or 1. Figure S1a shows the results from fitting logistic regressions to the simulated data sets. Figure S1b shows the results from fitting RFPM to the simulated data sets. Diamonds denotes the true log-odds ratios under the simulation model. [file 1756-0381-7-2-S1.zip › 7341464451022578_add2.tiff]
